# LGBTQ-Inclusive Hospice and Palliative Care: A Practical Guide to Transforming Professional Practice

**DOI:** 10.5195/jmla.2019.766

**Published:** 2019-10-01

**Authors:** Paul M. Blobaum

**Affiliations:** University Library, Governors State University, University Park, IL, pblobaum@govst.edu

This book is an important contribution to the literature of patient care of lesbian, gay, bisexual, transgender, and queer (LGBTQ) people and their loved ones, providing a practical framework for delivery of hospice and palliative care for all people. In her introduction, which can be downloaded for free from the publisher’s website, Kimberly D. Acquaviva notes many seminal textbooks on hospice and palliative care but observes that, at the most, the LGBTQ community is dealt with in a stand-alone chapter or otherwise in passing, if at all. This book fills a great need for in-depth discussion of this subject. The author’s main purpose is to remove social stigma and barriers to understanding the LGBTQ community and shift the paradigm to a focus on the care of the patient as a person with a family and friends, not a category.

The book is written as a textbook for nursing, medicine, chaplaincy, and social work students, with bullet points, case studies, and relevant standards throughout, but the book would be very useful beyond these disciplines. Everything about hospice and palliative care—from intake and care planning to death, and burial, and bereavement care—is addressed. Chapters follow the outline of objectives, key terms, chapter summaries, perspectives: personal stories from care givers and families, key points to remember, discussion questions, and a chapter activity that is geared toward integration of the chapter contents through reflection or application. An extensive glossary is helpful, though oddly, the terms “cisgender” and “queer” are not listed.

The word “inclusive” in the title is relevant to the scope of the book: to bring knowledge and awareness of this community to the forefront and to make all patient care inclusive. Chapter 1, “Self-Awareness and Communication,” can also be downloaded for free from the publisher and would be wonderful required reading for any health and human services student. In this chapter, the caregiver is invited to inventory personal feelings and beliefs, plan for the caregiving relationship, and develop a mitigation plan for issues that could stand in the way of delivering the best care possible, using the mnemonic device “CAMPERS,” which stands for clear purpose, attitudes and beliefs, mitigation plan, patient emotions, reactions, and strategy.

Most of the book is directed to the health care provider as an individual person, but the last chapter, Chapter 10, addresses the critical need to create and strengthen inclusive institutions. Excellent guidance is given for reviewing and writing inclusive institutional statements and policies by creating a platform with four planks: (1) an inclusive nondiscrimination statement; (2) provision of inclusive employee benefits, orientation, and training; (3) inclusive intake forms and processes; and (4) inclusive marketing and community engagement.

Overall this book is excellent, but for any future revisions, Chapter 7, “Ethical and Legal Issues,” would benefit from additional attention to legal matters with guidance on readings for more information. For example, in the brief discussion of do not resuscitate (DNR) orders, an important advanced directive, there is no mention of the probability that a separate DNR must be written for the hospital, for ambulance and medical transport, and again for the home health care provider or long-term care provider, something that would be important for the advocacy and education of the patient and family and friend caregivers of a person entering end-of-life care.

The author is a tenured faculty member at the George Washington University School of Nursing and self identifies as a member of the LGBTQ community, providing valuable expertise and insight via professional experiences as a nurse and personal experiences as a cancer patient in the health care system. The author enlisted the assistance of a panel of expert content reviewers to ensure the accuracy and completeness of the content. This book is recommended for academic and hospital libraries, and is essential reading for administrators in hospital, home health care, and long-term care settings. The book would be appropriate for a book club and should be considered for acquisition by public libraries with health care and consumer health collections.

***Paul M. Blobaum, MA, MS****, pblobaum@govst.edu, University Library, Governors State University, University Park, IL*

